# Exome sequencing identified a novel HIST1H1E heterozygous protein‐truncating variant in a 6‐month‐old male patient with Rahman syndrome: A case report

**DOI:** 10.1002/ccr3.5370

**Published:** 2022-02-07

**Authors:** Subba Rao Indugula, Sofia Saenz Ayala, Francesco Vetrini, Alyce Belonis, Wenying Zhang

**Affiliations:** ^1^ Division of Human Genetics Cincinnati Children’s Hospital Medical Center Cincinnati Ohio USA; ^2^ Department of Medical and Molecular Genetics Undiagnosed Rare Disease Clinic Indiana University School of Medicine Indianapolis Indiana USA; ^3^ Department of Pediatrics University of Cincinnati College of Medicine Cincinnati Ohio USA

**Keywords:** developmental delay, *HIST1H1E*, macrocephaly, Rahman syndrome

## Abstract

Rahman syndrome is a rare congenital anomaly syndrome recently described, which results from pathogenic variants in the HIST1H1E gene. The condition is characterized by variable somatic overgrowth, macrocephaly, distinctive facial features, intellectual disability, and behavioral problems. This report extends the genotype and clinical phenotype of HIST1H1E‐associated Rahman syndrome.

## Graphical abstract

Patient's facial images (A) at 5 weeks of age, (B) at 8 weeks of age, and (C) at 9 months of age

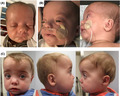

## 1 INTRODUCTION

Rahman syndrome is a rare autosomal‐dominant intellectual disability syndrome caused by monoallelic pathogenic frameshift variants affecting the C‐terminal domain (CTD) of the *HIST1H1E* gene. *HIST1H1E* is a single‐exon, intronless gene located on chromosome 6p22.2. It encodes the linker histone H1.4, a member of the H1 histone family, and functions as a structural component of chromatin to control DNA compaction, gene expression regulation, DNA replication, recombination, and repair. The HIST1H1E protein contains an N‐terminus region enriched in basic amino acids, a central conserved globular H15 domain involved in DNA binding, and a long C‐terminal tail enriched in lysine, serine, and proline.^1^

This condition, also known as *HIST1H1E* syndrome, was first described in 2014 and is characterized by overgrowth, distinctive facial features (full cheeks, high frontal hairline, hypertelorism, telecanthus, deep‐set eyes, downslanting palpebral fissures, micrognathia, etc.), intellectual disability (mostly moderate severity), and behavioral problems. Other symptoms include strabismus, hypothyroidism, cryptorchidism, skeletal and cardiac anomalies, abnormal dentition, hypotonia, and abnormal brain MRI with corpus callosum abnormalities being the most frequent finding.^2^, ^3^ Additionally, autism spectrum disorder has been described as a part of the presentation in a patient with similar facial features and significant intellectual disability.^4^

To date, there are 48 individuals affected with Rahman syndrome and 20 pathogenic protein‐truncating variants in the CTD in *HIST1H1E* reported globally. All reported cases of Rahman syndrome with parental analyses are *de novo*. The previously reported 20 CTD pathogenic variants are clustered to a 99‐bp region in the C‐terminal domain that is involved in chromatin binding and protein‐protein interactions. They result in the same shift in the reading frame and are predicted to generate similar truncated proteins, with a reduced net charge compared to the wild‐type protein, which is less effective in neutralizing negatively charged linker DNA.^3^ Functional characterizations of the aberrant C‐terminal tail of HIST1H1E have shown that it resulted in stable proteins that disrupted proper compaction of DNA and were associated with altered methylation pattern.^5^, ^6^ A direct link between aberrant chromatin remodeling, cellular senescence, and accelerated aging revealed a new feature of premature aging within affected individuals. Here, we describe another novel frameshift mutation in CTD in *HIST1H1E* in an infant with Pierre Robin sequence and central sleep apnea with Rahman syndrome.

## 2 CASE PRESENTATION

This is a 6‐month‐old male infant, dichorionic diamniotic twin, who was the second child to a nonconsanguineous Caucasian family. He was born at 35 weeks and 4 days gestation via cesarean section, following a pregnancy complicated by twin gestation, maternal preeclampsia, and transverse position of one twin sibling. His birthweight was 2.68 kg, and he was appropriate for gestational age (AGA), similar to his twin sister. Apgar scores were 8 and 9 at the first and fifth minutes after birth, respectively, and he had an uncomplicated nursery stay. He passed his hearing screen bilaterally, and his newborn screen was unremarkable.

**FIGURE 1:**
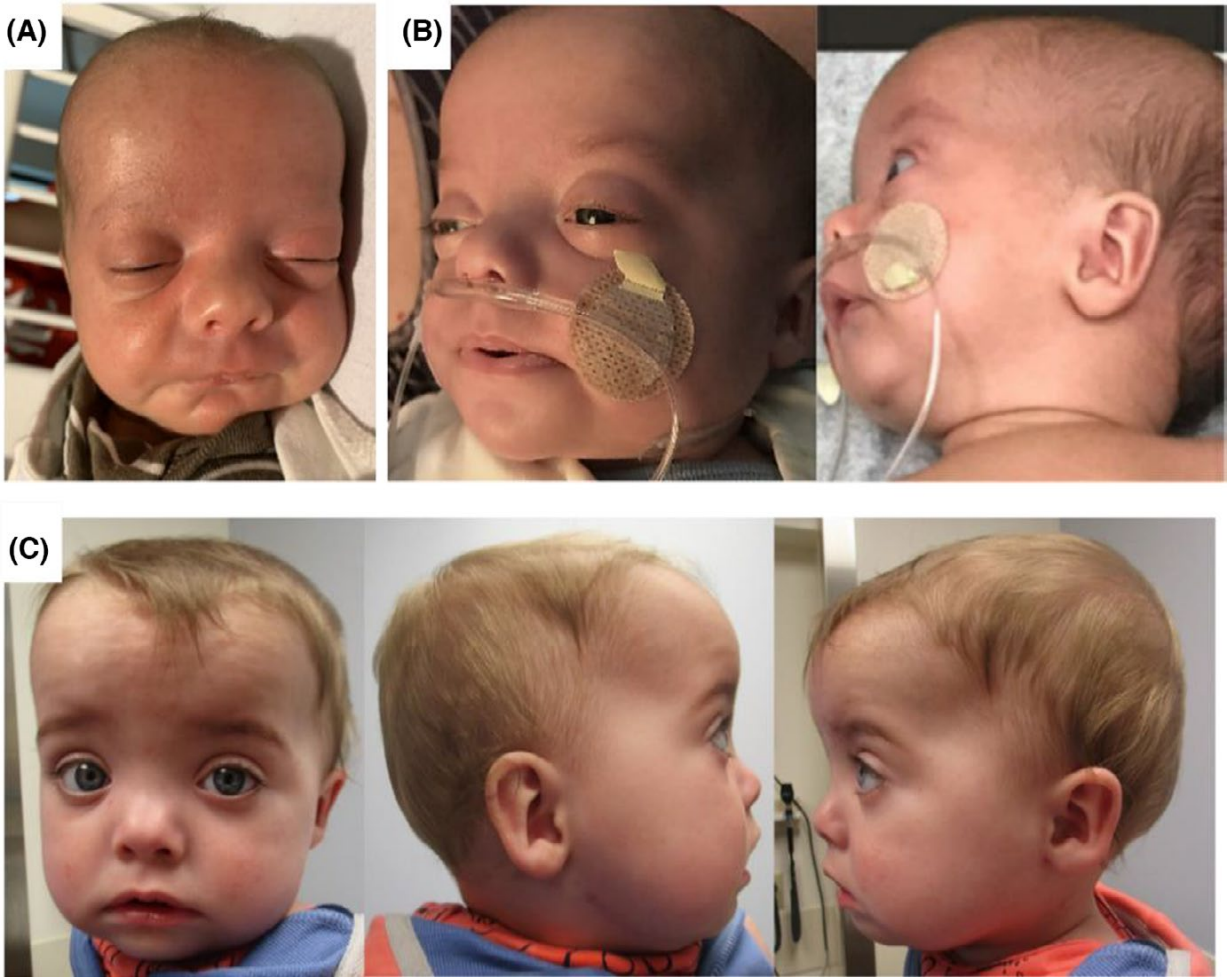
(A) At 5 weeks of age: Dysmorphic features include frontal bossing with broad forehead, hypertelorism, downslanting palpebral fissures, bulbous nasal tip, full cheeks, high frontal hairline, retrognathia, and micrognathia (B) At 8 weeks of age: status postbilateral mandible osteotomies and internal distractor placement (C) at 9 months of age

At 5 weeks of age, he was presented to the emergency room for spells of color change, breath holding, and abnormal eye movements. Spells were reported to occur mostly when transitioning from sleeping to waking, and he would recover spontaneously after a few seconds but appeared drowsy. His physical examination was remarkable for relative macrocephaly with head circumference at the 91st percentile, while weight and length were at the 20th‐40th percentile, 1/6 systolic heart murmur, and dysmorphic features including mild micrognathia, retrognathia, frontal bossing, broad forehead, hypertelorism, bulbous nasal tip, full cheeks, and high frontal hairline (Figure 1). He underwent a throughout evaluation and work‐up including normal video electroencephalogram without evidence of seizures, normal chest X‐ray, normal head ultrasound, and normal brain MRI. Polysomnography revealed severe obstructive sleep apnea with apnea‐hypopnea index [AHI] of 34.85 per h and obstructive index of 31.26 per h, with the lowest oxygen saturation being 90%, which improved partially on supplemental oxygen of 0.25 liters via nasal cannula. There was no evidence of hypoventilation on pre‐ and post‐blood gases. An airway evaluation including microlaryngoscopy and bronchoscopy demonstrated moderately dynamic collapse of the bilateral arytenoids with glossoptosis, mild tracheomalacia and laryngomalacia, and submucous cleft palate.

Genetic testing included normal SNP microarray and abnormal whole‐exome sequencing trio, which identified a *de novo* heterozygous pathogenic variant, c.505_506insT (p. Lys169IlefsTer27), in the *HIST1H1E* gene associated with Rahman syndrome. More details about methodology of testing and results are included below under the molecular analysis and results sections. Additional work‐up given this genetic diagnosis included an echocardiogram that identified trivial mitral valve stenosis. He was referred to an early intervention program and started physical therapy to address his delayed motor milestones.

At 7 weeks of age, he underwent mandibular osteotomies with application of internal mandibular distractors bilaterally given his diagnosis of Pierre Robin sequence (Figure 1B‐C). Repeat polysomnography at 5 months old following distraction showed central sleep apnea (central apnea index of 11.4 per h) and mild obstructive sleep apnea (obstructive apnea‐hypopnea index of 3.1 per h), without evidence of hypoventilation. Recommendation was to continued oxygen supplementation at 0.5 liters via nasal cannula. At his 9‐month‐old follow‐up (Figure 1), he was noted to have global developmental delay and mild axial hypotonia. He had good head control, and he could roll over, but he was still not sitting unsupported, crawling, or pulling to a stand; he was able to transfer objects between hands and was working on pincer grasp; he could wave “bye‐bye” and was very attentive of his environment; he could laugh, squeal, and coo, but he was not babbling yet. He was enrolled in physical and speech therapies and was steadily achieving new milestones. His growth was appropriate for his age with his weight in the 59th percentile, length in the 14th percentile, and head circumference in the 99th percentile consistent with macrocephaly. His most recent sleep study demonstrated severe degree of mixed sleep‐disordered breathing, with primarily central sleep apnea characterized by a periodic breathing pattern, partially corrected on oxygen at 1/8 liter via nasal cannula. No reportable variants were identified in genes associated either with obstructive sleep apnea or central sleep apnea in his exome sequencing. Also, as this case was the first one to report Pierre Robin sequence as an additional phenotype in Rahman syndrome, we analyzed all the genes associated with Pierre Robin sequence and found no reportable variants in addition to a previously normal SNP microarray, hence ruling out other known genetic causes for Pierre Robin sequence in this patient. Family history was unremarkable, and his twin sister was reported to be smaller compared to the proband in all growth parameters despite her birthweight being appropriate for gestational age, and her development is on track and even advanced in motor skills.

## 3 MOLECULAR ANALYSIS

Whole‐exome sequencing was performed on genomic DNA of the patient, and his parents using the SureSelect CREv1 (Agilent Technologies, CA) targeted sequence capture method to enrich the whole exome. The exome was sequenced using an Illumina sequencing system with paired‐end reads with more than 95% of the target regions at a minimum coverage of X 20. The exome sequence reads were mapped and compared to human genome build UCSC hg19 reference sequence. Variants within coding exons, flanking sequences, and CREv1‐targeted promoter/deep intronic HGMD known pathogenic variants were evaluated by an in‐house developed bioinformatic analysis pipeline that includes the usage of Burrows‐Wheeler aligner (BWA) and genome analysis toolkit (GATK). Variants identified by the GATK‐based bioinformatic pipeline were uploaded to the Fabric Genomics Analysis app (Fabric Genomics, Inc.), which was used to annotate, analyze, and classify the identified variants. Clinical significance of variants was assessed based on the standards and guidelines for the interpretation of sequence variants, recommended by ACMG‐AMP.^7^ Sanger sequencing was performed to confirm the reported variant.

## 4 RESULTS

Exome sequencing on the proband and his parents identified a novel, 1‐base pair insertion variant, c.505_506insT, in the *HIST1H1E* gene. This results in a frameshift in the CTD, starting with codon Lysine 169, changing this amino acid to an Isoleucine residue, and creating a premature stop codon at position 27 of the new reading frame, denoted p. Lys169IlefsTer27. Trio analysis found this variant to be *de novo* in the proband, and the results were confirmed by Sanger sequencing. This variant has not been reported previously in the literature, to our knowledge. This variant is absent from the population database, Genome Aggregation Database (gnomAD), and no minor allele frequency (MAF) is available. This variant is predicted to cause loss of normal protein function through protein truncation by replacing the last 51 amino acids of the HIST1H1E protein with 26 incorrect amino acid residues, of which the last 25 amino acids share exactly the same sequence with those in all 20 CTD pathogenic variants reported with Rahman syndrome^3^, ^5^, ^6^ (Figure 3).

## 5 DISCUSSION

*HIST1H1E*‐associated Rahman syndrome has been identified in 48 individuals, with 20 pathogenic protein‐truncating variants in CTD in *HIST1H1E* reported globally. Here, we present another novel frameshift variant in CTD in *HIST1H1E* identified through whole‐exome sequencing in an infant with features not previously described with Rahman syndrome, including Pierre Robin sequence with submucous cleft palate and central sleep apnea. In addition to these findings, he shared similar dysmorphic facial features, developmental delay, and macrocephaly as other patients described formerly.^2^, ^3^, ^8^ This infant underwent mandibular distraction with improvement in obstructive sleep apnea, although central apnea worsened. We hypothesized that his hypotonia is the major contributing factor to his central apnea, and it is expected to improve over time as his hypotonia improves. Although cardiac abnormalities were reported in 43% of patients in one cohort,^2^ mitral valve stenosis was not described before. Similarly, common facial features in Rahman syndrome include full cheeks, high hairline, downslanting palpebral fissures, and hypertelorism, but Pierre Robin sequence with submucous cleft palate has not been described before. HIST1H1E as a member of the family of H1 linker histone proteins contributes to the organization and stabilization of chromosomal DNA, in addition to the folding of nucleosome filaments into higher‐order structures and modulation of gene expression and chromatin remodeling. Several studies have also proven that germ cell‐specific H1 subtypes seem to play an important role in early embryogenesis and reprogramming,^9^ which could contribute to the facial abnormalities described in individuals with aberrant *HIST1H1E*. Pierre Robin sequence and cleft palate are common features in chromatin remodeling disorders, but more cases are needed to determine the specific genes regulated by *HIST1H1E* that may play an important role in facial development.

**FIGURE 2:**
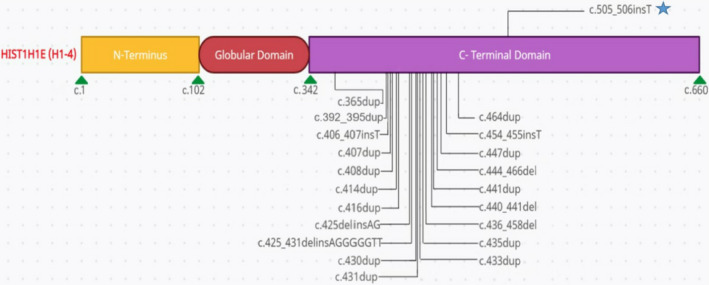
Schematic diagram of *HIST1H1E* pathogenic variants Previously reported 20 CTD pathogenic variants, shown below the diagram, are clustered to a 99‐bp region in the C‐terminal domain. The novel variant detected in this case, shown above the diagram and highlighted with a star, is located 42‐bp downstream of the reported to date most 3' pathogenic variant associated with Rahman syndrome

**FIGURE 3:**
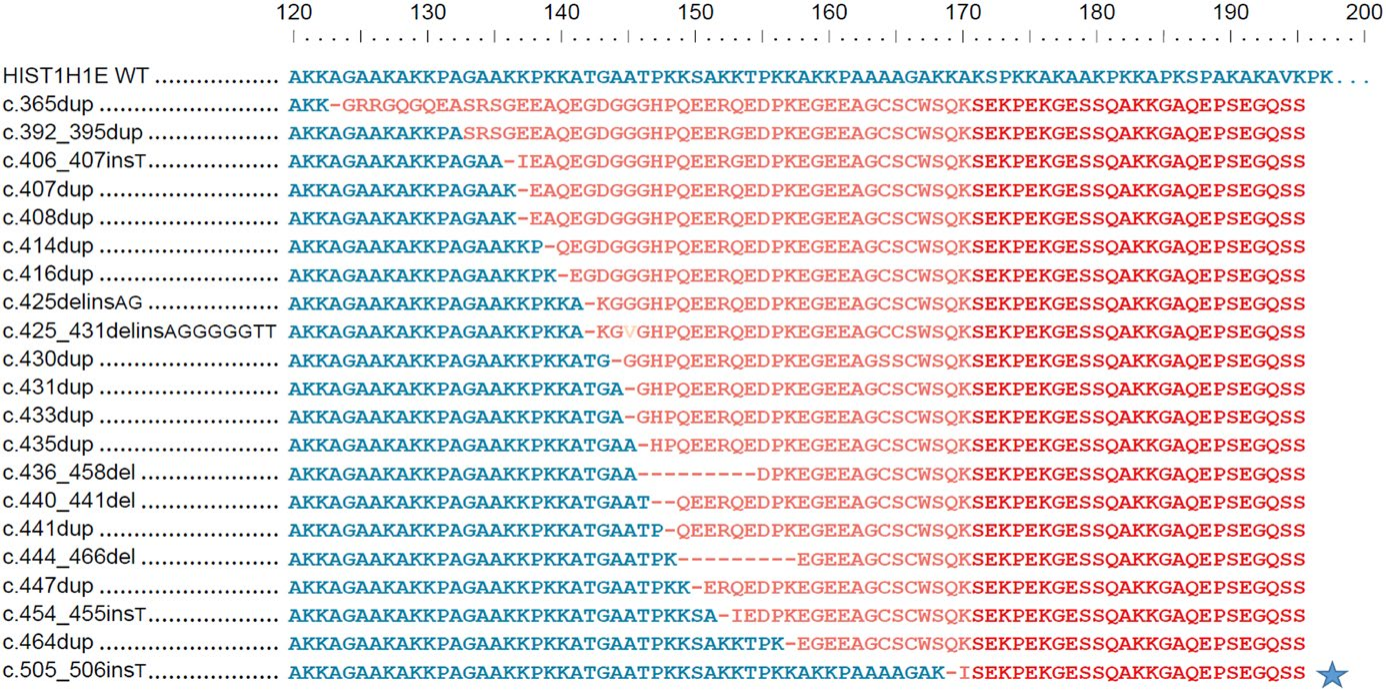
Amino acid sequence alignment of *HIST1N1E* pathogenic variants All top 20 previously reported CTD pathogenic variants share the same last 38 amino acids in the C terminus. The novel variant detected in this patient, c.505_506insT, creates a new 26 AA sequence, and the last 25 (highlighted in red) are shared with other pathogenic variants

The previously reported 20 CTD pathogenic variants are clustered to a 99‐bp region in the C‐terminal domain (Figure 2). The novel variant, c.505_506insT (p. Lys169IlefsTer27), detected in this case is located 42‐bp downstream of the reported to date most 3’ pathogenic variant associated with Rahman syndrome, further extending the range of the cluster. This variant has not been reported previously in literature and is absent from the population database (gnomAD), and no minor allele frequency (MAF) is available. All 20 CTD pathogenic variants share the same last 38 amino acids in the C terminus, while this novel variant is predicted to cause protein truncation by replacing the last 51 amino acids of the HIST1H1E protein and creating a new 26‐amino acid sequence, of which the last 25 are shared with other pathogenic variants^3^, ^5^, ^6^ (Figure 3).

Protein‐truncating variants in the intronless histones have been shown to evade nonsense‐mediated mRNA decay. All previously reported pathogenic variants, including this novel variant in the *HIST1H1E* gene, result in the same shift in the reading frame and are predicted to generate similar truncated proteins, with a reduced net charge compared to wild‐type proteins. The mutant protein is, therefore, likely to be less effective in neutralizing negatively charged linker DNA and in limiting DNA binding and protein‐protein interactions.^3^ Functional characterizations of these aberrant C‐terminal tails of HIST1H1E have shown that it results in stable proteins that disrupt proper compaction of DNA and are associated with altered methylation pattern.^5^, ^6^ An expression study performed on one of the previously reported pathogenic variant, c.435dup or p. Thr146Hisfs*50, demonstrates reduced protein expression, which suggests that haploinsufficiency of HIST1H1E protein, and loss of function could also be the underlying mechanism of brain dysfunction leading to the neurodevelopmental phenotype, such as intellectual disability and autism.^4^

The present report further extends the genotype and the clinical phenotypes of *HIST1H1E*‐associated Rahman syndrome. This study supports that *HIST1H1E* should be considered for patients with intellectual disability and/or multiple congenital anomalies through single‐gene testing, multigene panel, or comprehensive genomic testing.

## CONFLICT OF INTEREST

The authors declare no potential conflict of interest.

## AUTHOR CONTRIBUTIONS

Conceptualization and design: Subba Rao Indugula, Sofia Saenz Ayala, and Wenying Zhang. Analysis and interpretation of data: Subba Rao Indugula, Francesco Vetrini, Sofia Saenz Ayala, Alyce Belonis, and Wenying Zhang. Drafting of the article: Subba Rao Indugula and Sofia Saenz Ayala. Critical revision of the article: Francesco Vetrini, Alyce Belonis, and Wenying Zhang. Study supervision: Wenying Zhang.

## 6 ETHICAL APPROVAL

None.

## CONSENT

The authors confirm that a signed consent from the patient's parent was obtained prior to publication.

## Data Availability

None.
